# Acetylation-Specific Interference by Anti-Histone H3K9ac Intrabody Results in Precise Modulation of Gene Expression

**DOI:** 10.3390/ijms23168892

**Published:** 2022-08-10

**Authors:** Simonetta Lisi, Matteo Trovato, Ottavia Vitaloni, Marco Fantini, Michele Chirichella, Paola Tognini, Sara Cornuti, Mario Costa, Marco Groth, Antonino Cattaneo

**Affiliations:** 1 Bio@SNS Laboratory, Scuola Normale Superiore, 56126 Pisa, Italy; 2 European Molecular Biology Laboratory, Genome Biology Unit, 69117 Heidelberg, Germany; 3 Department of Translational Research and New Technologies in Medicine and Surgery, University of Pisa, 56126 Pisa, Italy; 4 Institute of Neurosciences, Consiglio Nazionale Delle Ricerche, 56124 Pisa, Italy; 5 Leibniz Institute on Aging—Fritz Lipmann Institute (FLI), 07745 Jena, Germany

**Keywords:** intracellular antibodies, post-translational modifications (PTMs), epigenetic words, histone acetyltransferases HATs, HAT inhibitors (HATi)

## Abstract

Among Histone post-translational modifications (PTMs), lysine acetylation plays a pivotal role in the epigenetic regulation of gene expression, mediated by chromatin modifying enzymes. Due to their activity in physiology and pathology, several chemical compounds have been developed to inhibit the function of these proteins. However, the pleiotropy of these classes of proteins represents a weakness of epigenetic drugs. Ideally, a new generation of epigenetic drugs should target with molecular precision individual acetylated lysines on the target protein. We exploit a PTM-directed interference, based on an intrabody (scFv-58F) that selectively binds acetylated lysine 9 of histone H3 (H3K9ac), to test the hypothesis that targeting H3K9ac yields more specific effects than inhibiting the corresponding HAT enzyme that installs that PTM. In yeast scFv-58F modulates, gene expression in a more specific way, compared to two well-established HAT inhibitors. This PTM-specific interference modulated expression of genes involved in ribosome biogenesis and function. In mammalian cells, the scFv-58F induces exclusive changes in the H3K9ac-dependent expression of specific genes. These results suggest the H3K9ac-specific intrabody as the founder of a new class of molecules to directly target histone PTMs, inverting the paradigm from inhibiting the writer enzyme to acting on the PTM.

## 1. Introduction

Among diverse histone post-translational modifications (PTMs), histone acetylation of lysine residues plays a pivotal role in the epigenetic regulation of gene expression [1,2,3]. Histone acetylation is controlled by two main enzymatic classes, the HATs and the HDACs [4], and it also promotes transcription by providing binding sites for bromodomain-containing proteins [5]. The functional study and validation of individual PTM targets presents formidable challenges, and can only be indirect. Indeed, PTMs cannot be selectively inhibited by nucleic-acid-based interference approaches (such as, for instance, RNA interference) because they interfere with all variants of a protein simultaneously. A number of small molecules targeting Histone Deacetylases (HDACs), Histone Acetyl-Transferases (HATs) [6,7,8,9,10], as well as Bromodomains [11], are available, some of which are undergoing clinical trials for oncological and neurological pathologies [12,13]. However, these chemical inhibitors show generally poor target selectivity and unpredictable pleiotropic effects [14].

At present it is not straightforward to select specific small molecules inhibiting only a defined subset of histone modifying enzymes. Moreover, each such enzyme modifies multiple residues on different proteins. For this reason, HDAC, HAT, or BET inhibitors are intrinsically pleiotropic. Besides histones, a variety of non-histone substrates have been shown to be acetylated by HATs, thus the HATs are now generally categorized as lysine acetyltransferases [15].

In order to study the effects of a single epigenetic PTM on cell function [16,17] and to obtain a more focused therapeutic outcome, it would be important to interfere precisely with a single acetylated residue. Thus, developing compounds that can selectively target PTMs, as opposed to inhibiting the modifying enzymes, represents a ‘holy grail’ of drug development in different fields, including epigenetic therapeutics, cancer, and neurodegenerative diseases. 

To this aim, in this paper, we exploited intracellularly expressed antibody domains (intrabodies) [18,19,20,21] and the recently developed P.I.S.A (*Post-translational Intracellular Silencing Antibody*) technology [22] for the in vivo selection of intrabodies that specifically bind PTM-epitopes on the protein target. The strategy of directly targeting single epigenetic marks with PTM-specific intrabodies is predicted to achieve a superior specificity and selectivity with respect to the current approaches that target instead the epigenetic modifying enzymes responsible for installing the PTM [23]. However, a direct comparison between the two strategies is lacking.

Here, we tested this prediction by exploiting a specific anti-H3K9ac single-chain variable fragment (scFv) intrabody (scFv-58F), previously shown to induce a functional in-cell interference of the acetylated H3, by selectively binding to K9 acetylated histones H3 [22]. We demonstrated that targeting directly site-specific acetylated histone H3 with scFv-58F affects gene expression in a more specific and subtle way, compared to two well characterized HAT inhibitors (HATis). This H3K9ac-specific protein interference allowed for the identification of a group of genes involved in ribosome biogenesis and function as a target for the specific transcriptional regulation by H3K9 acetylation.

## 2. Results

### 2.1. The Transcriptional Effects of the Anti-H3K9ac scFv-58F Intrabody in Comparison to Those Induced by HAT Inhibitors

We compared the global gene expression changes driven by the histone PTM-selective interference with those induced by the inhibition of the corresponding modifying enzymes. We chose site-specific histone H3 acetylation as a target for this comparison, and we investigated the transcriptomic response in yeast cells, either expressing the intrabody anti-H3K9ac scFv-58F [22] or treated with HATs inhibitors [13]. The anti-H3K9ac scFv-58F is a recombinant antibody domain selected via P.I.S.A technology to bind acetylated lysine 9 on yeast and mammalian histone H3. When expressed in cells, the anti-H3K9ac scFv-58F specifically binds acetylated K9 on histone H3 [22]. For this comparative study, we used two chemical HAT inhibitors (HATi): CPTH2, a synthetic inhibitor of the Gcn5 HAT [24], responsible for H3K9 acetylation [25], and Curcumin, a natural compound, undergoing clinical trials [26], reported to inhibit p300/CBP HAT activity, but for which also non-HAT targeted activities are reported [27].

Three yeast strains were used: the pL220–58F–HA strain, stably expressing the scFv-58F-HA, the pL220–645–HA strain expressing an unrelated intrabody scFv-645-HA (an anti-neuroligin-2 intrabody), and a pL220–HA strain, carrying the empty vector pL220. 

Inhibition of histone H3 acetylation was obtained by treating the control yeast strain pL220–HA with either Curcumin (200 µM) or CPTH2 (400 µM) in SD-L media for 1 h (see Figure S1). The baseline control was represented by pL220–HA cells treated with the same concentration of DMSO (0.4%) used to dissolve the drugs. 

Total RNA was extracted from pL220–HA grown either in the presence of Curcumin or CPTH2, as well as from pL220–HA, pL220–58F–HA, or pL220–645–HA (the two intrabody-expressing strains) all grown in DMSO treated media, and RNA samples underwent RNA-sequencing (RNA-seq) analysis. Differential gene expression (DEG) analysis was performed, comparing either the two HATi-treated or the two intrabody-expressing conditions against the DMSO-treated baseline control condition (see Dataset S1).

**Figure 1:**
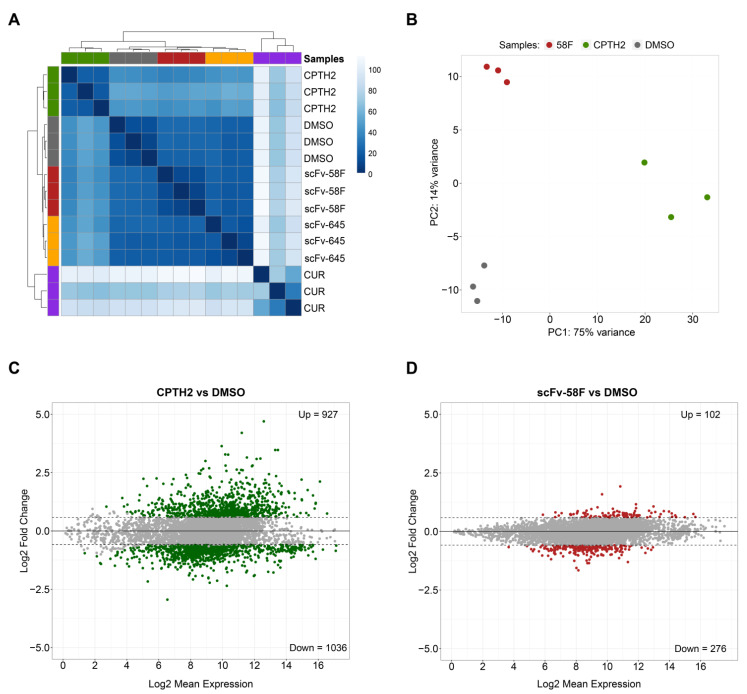
Expression of scFV-58F intrabody induces fewer transcriptional changes than treatment with HAT inhibitors. (**A**) Hierarchical clustering of RNAseq data from three biological replicates for each analyzed condition in yeast cells (CUR = curcumin-treated cells, CPTH2 = CPTH2-treated cells, DMSO = DMSO-treated cells, 58F = cells expressing scFv-58F, 645 = cells expressing scFv-645) (**B**) Principal Component Analysis (PCA) of the RNAseq data from three experimental conditions 58F, CPTH2, and DMSO (negative control). For clarity, Curcumin-treated samples (which account for most of the variation) and the scFv-645 samples were omitted in this PCA plot and are reported in Supplementary Figure S2A. Among the remaining nine samples, the principal component of variation is accounted for by CPTH2 samples (75% of variance explained by the first principal component, i.e., PC1). (**C**,**D**) MA plot (i.e., log2 fold-changes on y-axis versus log average expression signal on x-axis) of the significantly differentially expressed genes (FDR = 0.05), for CPTH2-treated cells vs. the DMSO-treated control cells (**C**) and for scFv-58F expressing cells vs. the DMSO-treated control cells (**D**). The number of significantly up-/down-regulated genes (p.adj < 0.05 & FC cutoff = 1.5) is reported for both comparisons.

Hierarchical Clustering and Principal Component Analysis (PCA) of the RNA-seq data showed that the Curcumin and CPTH2 treated samples account for most of the variation (Figure 1A and Figure S2A), with the two intrabody-expressing conditions being closest to the DMSO-treated control samples (Figure 1A,B and Figure S2A). Despite being close to DMSO-treated controls, the scFv-58F and scFv-645 samples formed two clearly separate clusters, indicating a different impact on global gene expression induced by the expression of the two intrabodies (Figure 1A and Figure S2D).

In line with the expected pleiotropy of the HATi drugs, both Curcumin and CPTH2 treatments broadly affected gene expression in *S. cerevisiae* to a much greater extent, inducing deregulation of 3703 and 1963 genes (p.adj < 0.05 and fold-change cutoff = 1.5), respectively (Figure 1C and Figure S2B). On the other hand, the scFv-58F intrabody induced less dramatic transcriptomic changes (378 DEGs with p.adj < 0.05 and fold-change cutoff = 1.5. Figure 1D). Despite the lower number of DEGs in the intrabody expressing strains (Figure 1D for scFv-58F and Figure S2C for the negative control non PTM-targeting intrabody scFv-645), expression of scFv-58F still led to deregulation of a discrete set of genes. Notably, 73% (i.e., 276 out of 378) of the genes affected by scFv-58F expression were downregulated. This is in line with the selective interference of the intrabody with the H3K9ac modification, which usually marks active promoters. The set of DEGs in the scFv-58F-expressing cells provide, therefore, a transcriptional fingerprint of the effects of specifically blocking the H3K9ac epigenetic mark in yeast cells.

### 2.2. A Large Fraction (~70%) of the Genes Specifically Modulated by the scFv-58F Intrabody Are Also Modulated by CPTH2 Treatment

**Figure 2:**
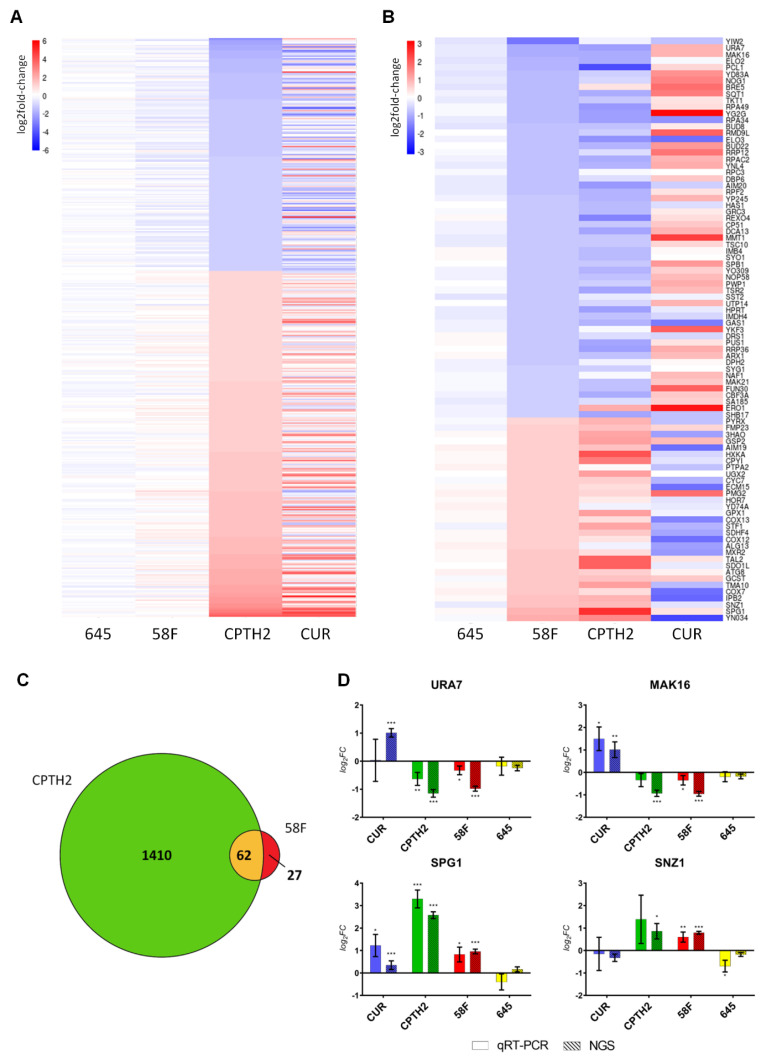
The expression of scFv-58F intrabody modulates a small but largely overlapping subset of genes altered by HATis. (**A**) Heatmap of mRNAs (*n* = 435) that are significantly modulated after CPTH2 treatment. mRNAs are sorted by log2FC in CPTH2 vs. DMSO: expression patterns in the presence of CPTH2 and Curcumin show overall strong similarity. Statistical constraints: FC cut-off = 2; p.adj(CPTH2 vs. DMSO) < 0.05; p.adj(scFv-645 vs. DMSO) > 0.05. (**B**) Heatmap of mRNAs (*n* = 89) that are significantly modulated by scFv-58F. The genes modulated by the control intrabody scFv-645 were subtracted (see statistical constraints below). mRNAs are sorted by log2FC in scFv-58F vs. DMSO: expression patterns in the presence of scFv-58F and CPTH2 treatment are similar, while the expression pattern is opposite with Curcumin treatment. Swiss-Prot Entry names were provided in the heatmap where primary gene names were not available. Statistical constraints: FC cut-off = 1.5; p.adj(scFv-58F vs. DMSO) < 0.05; p.adj(scFv-58F vs. scFv-645) < 0.05; p.adj(scFv-645 vs. DMSO) > 0.05. (**C**). Venn diagram showing the overlap between DEGs in scFv-58F and CPTH2 treatments vs. DMSO (FC cutoff = 1.5). (**D**) Validation through RT-qPCR of selected candidate mRNAs identified from RNAseq data. Log2FC +/− SD values of real-time PCR (solid color) and RNAseq data (striped) for the four chosen differentially expressed genes in the presence of scFv-58F. Values for each treatment are representative of *n* = 3 biological replicates. For real-time PCR data: ΔCt = Ct GENE - Ct TUB1. log2(FC) = −ΔΔCt normalized on tubulin (TUB1) and DMSO control samples. SD = 2 var∆Ct DMSO + (var∆Ct TREATMENT), where “TREATMENT” represents each of the four different experimental conditions (CUR; CPTH2; scFv-58F; scFv-645). Student t-test (TREATMENT vs. DMSO, two tails) was performed on ΔCt values. Calculated *p* values were adjusted with the Benjamini–Hochberg procedure; the same statistical correction was applied for RNAseq data; * p.adj < 0.05, ** p.adj < 0.01, *** p.adj < 0.001.

To further investigate the impact of the two HATi on gene expression, we selected genes that are both significantly differentially modulated in the presence of CPTH2 treatment (p.adj < 0.05 and fold-change cutoff = 2) and not affected in the presence of the non-PTM targeting intrabody scFv-645 (in order to account for non-specific gene expression changes due to the expression of an intracellular antibody in yeast cells). This set of differentially expressed genes was ordered by increasing log2 fold change in the heat-map shown in Figure 2A. This analysis revealed that the global gene expression pattern induced by CPTH2 and Curcumin shows an overall similarity (Figure 2A, Dataset S2). As displayed in the heatmap, this set of genes (*n* = 435) was only modestly affected in the scFv-58F condition.

Next, we defined a subset of genes specifically modulated by scFv-58F, by selecting those genes that are significantly differentially expressed in the presence of the H3K9ac-targeting intrabody (p.adj < 0.05 and fold-change cutoff = 1.5) and subtracting those that are modulated by the non-specific control intrabody scFv-645. This leads to a list of 89 genes whose expression is specifically modulated by the scFv-58F intrabody. Notably, the overall expression pattern of the 89 genes defined in this way, was strikingly similar between the scFv-58F and CPTH2 conditions (Figure 2B). In particular, a large fraction (i.e., 62/89, ~70%) of the genes specifically modulated by scFv-58F was also modulated and differentially expressed after CPTH2 treatment (Figure 2C).

Furthermore, for this set of genes the expression pattern observed after Curcumin treatment appears to be reversed, if compared to scFv-58F and CPTH2 treatments (Figure 2B). This is most likely due to the fact that not all actions of Curcumin on gene expression are ascribable to HAT inhibition, but also to some other known opposite effects on HDACs [28] and on other non-histone targets [29]. The expression of scFv-58F alters a subset of genes that constitute only one branch of the targets of the CPTH2 HATi drug, de facto pruning all the other undesired (i.e., non H3K9ac-dependent) transcriptional side-effects. Moreover, despite CPTH2 and Curcumin having a very similar overall effect on the cell, based on global transcriptional patterns, they differ in this particular subset modulated by the intrabody scFv-58F, namely the genes regulated by H3K9 acetylation, in which they demonstrated an opposite effect. This fact corroborates the idea that the effect of these HATi drug types is pleiotropic and modular and that we can directly affect a single branch of their targets with intracellular antibodies interfering with one specific PTM target.

From these results we conclude that the transcriptional effect obtained by a direct interference with H3K9ac, using an H3K9ac-selective intrabody, is much more defined and restricted, when compared to that obtained with HATi treatment, which results instead in a much broader transcriptional outcome.

A subset of the genes most strongly modulated by scFv-58F was selected for validation by qPCR (see Figure 2B): we selected the two top upregulated (i.e., URA7, MAK16) and the two top downregulated genes (i.e., SPG1 and SNZ1) by scFv-58F expression (Figure 2D). Notably, qPCR data (solid columns) replicated the results obtained by NGS (striped columns), with a high grade of statistical significance (Figure 2D).

### 2.3. Gene Ontology Enrichment Analysis Links H3K9ac-Specific Interference to Transcriptional Regulation of Ribosome-Related Genes

**Figure 3:**
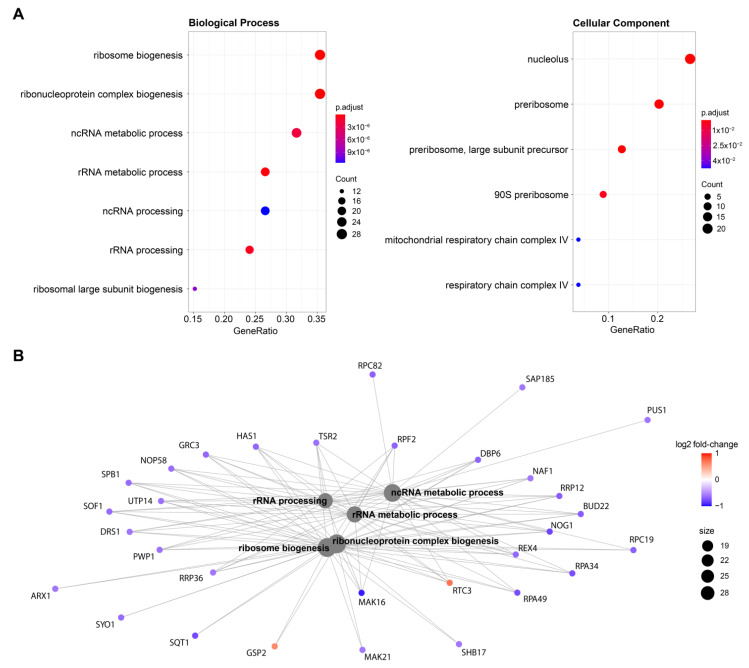
Gene ontology enrichment analysis (**A**) Gene ontology enrichment analysis of the genes significantly modulated by scFv-58F expression. Dot plots depict the main significant terms for the categories “Biological Process” (**left**) and “Cellular Component” (**right**). (**B**) Cnet plot displaying genes involved in the gene-ontology significant terms, with log2 fold-change expression with respect to the control DMSO.

As far as the mechanism and the consequences of the H3K9ac-specific interference is concerned, inspection of the intrabody-specific differentially expressed genes by scFv-58F (removing the effect of the presence of a generic intrabody in the cell) shows that 65% of DEGs (*n* = 58) are downregulated (Figure 2B). This is in line with the selective interference by the intrabody with the acetylated H3K9, a PTM known to mark transcriptionally active promoters and hence generally associated with transcriptional activation [30]. Therefore, binding of the intrabody to H3K9ac may competitively hinder the access of other transcriptional regulators to this docking site on chromatin, thereby affecting transcription. Gene ontology enrichment analysis of the scFv-58F DEGs showed a prominent modulation of genes involved in ribosome biology (e.g., nucleolus, ribosome biogenesis, rRNA processing, Figure 3A), with the vast majority of those genes being downregulated by scFv-58F (Figure 3B). This indicates that the H3K9 acetylation might be specifically connected with the collective transcriptional regulation of genes involved in ribosome biogenesis and function. Notably, a similar link between H3K9 post-translational modifications and ribosome function in yeast was previously reported in histone mutagenesis studies [31] or via RNA-seq and ChIP-seq analysis [32,33].

### 2.4. Gene Expression in Mammalian Cells Expressing scFv-58F

The experiments in yeast cells provided a clear-cut demonstration that directly targeting single epigenetic marks with PTM-specific intrabodies achieves a superior specificity and selectivity with respect to targeting the modifying enzymes responsible for installing the PTM. Having investigated the transcriptional consequences of H3K9ac-specific interference in yeast cells, we aimed to explore if this PTM-specific intrabody approach is also effective in mammalian cells. 

Stably-transfected HeLa cells expressing HA-tagged scFv-58F were analyzed. Nucleosome-enriched protein extracts were prepared from HeLa cells expressing the scFv-58F intrabody targeted to the nucleus via a nuclear localization sequence (scFv-58F-NLS) or retained in the cytoplasm (devoid of the nuclear localization sequence (scFv-58F-Cyto)) and from untransfected HeLa cells (WT). The scFv-58F-Cyto format of the intrabody serves as a control for the subcellular specificity of the interaction of scFv-58F-NLS with K9 acetylated H3 in nucleosome-enriched protein extracts.

**Figure 4:**
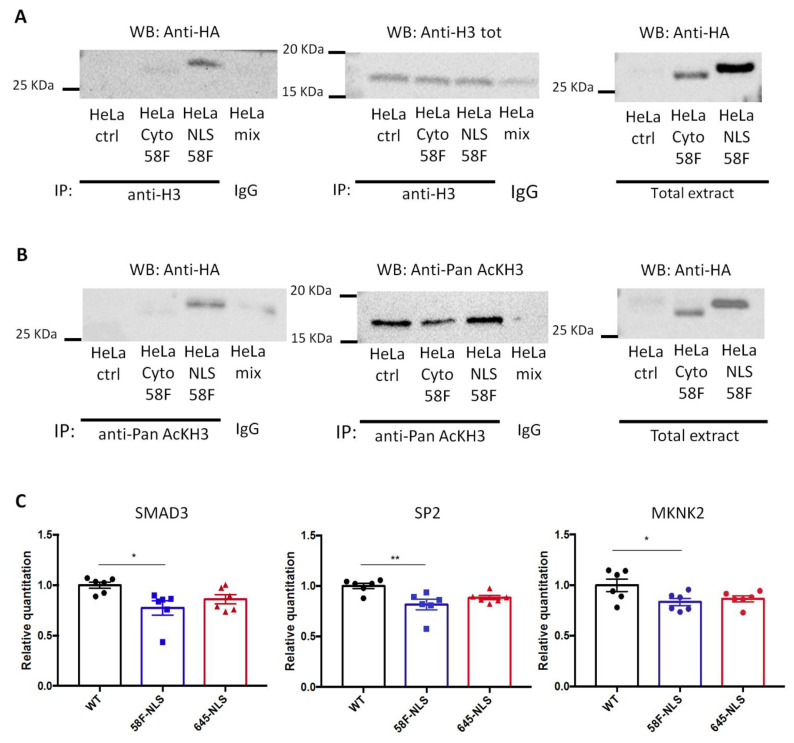
SscFv-58F expression in the mammalian system. (**A**) Stably transfected HeLa cells expressing HA tagged scFv-58F-NLS (HeLa NLS 58F), HA tagged scFv-58F-Cyto (HeLa Cyto 58F), and WT HeLa cells not transfected (as control, HeLa ctrl) were used in the experiment. Lysates enriched in nucleosomes were prepared and subjected to immunoprecipitation (IP) with anti-total Histone H3 antibody (Anti-H3 Tot). Immunoprecipitated proteins were detected by western blot (WB) using the anti-HA antibody (right blot) or anti-H3 Tot antibody (middle blot). On the right the input non immunoprecipitated extract was blotted with the anti-HA antibody. (**B**) The same stably transfected HeLa cells and WT HeLa cells were used for this IP experiment. The nucleosome enriched lysates were immunoprecipitated with an antibody against the pan acetylated Histone H3 (anti-Pan AcKH3). Immunoprecipitated proteins were detected by western blot (WB) using the anti-HA antibody (right blot) or the anti-Pan AcKH3 antibody (middle blot). On the right, the input extract not immunoprecipitated were blotted with the anti-HA antibody. HeLa mix: a mix of nucleosome extract prepared from HeLa transfected with scFv-58F-NLS (HeLa NLS 58F), HeLa transfected with scFv-58F-Cyto (HeLa Cyto 58F), and not transfected WT HeLa cells (HeLa ctrl) immunoprecipitated with a specific-antibody isotype matched control immunoglobulin (IgG). (**C**) RNA was prepared from WT HeLa cells (WT), transfected HeLa cells stably expressing scFv-58F-NLS (58F-NLS), and transfected HeLa cells stably expressing scFv-645-NLS (645-NLS). qPCR analysis of SMAD3, SP2, and MKNK2 genes was performed. Data is represented as relative RNA levels of SMAD3, SP2, or MKNK2 normalized to beta actin (ACTB gene). (*n* = 6 per groups, one-way ANOVA, post-hoc Tukey’s multiple comparison test: WT vs. 58F-NLS *p* < 0.05 *, *p* < 0.01 **. All other comparisons are not significant). Error bars represent SEM, dots over the histogram represent individual biological replicates.

To detect a physical association between scFv-58F and acetylated H3 histone, immunoprecipitation (IP) of acetylated H3 and of “total H3” (using an antibody whose binding to H3 is unaffected by the presence or not of the acetylation) was performed. Notably, both acetylated and total histone H3 IPs pulled down scFv-58F-NLS, as shown in the subsequent anti-HA blot (detecting the HA-tagged scFv-58F) (Figure 4A,B). The intensity of the scFv-58F bands on the western blot, after IP of acetylated H3 is equal to that of the band after IP of “total H3”, showing that the scFv-58F specifically interacts with the acetylated pool of H3. On the other hand, IP of acetylated and of total histone H3 from nucleosome-enriched protein extracts, did not pull down scFv-58F-cyto. Altogether, this demonstrates that the scFv-58F intrabody is able to interact with the acetylated H3 in the nucleus of mammalian cells, and the intrabody localized in the cytoplasm fails to bind acetylated H3 in nucleosome-enriched protein extracts.

Having established that scFv-58F binds acetylated histone H3 in the nucleus of mammalian cells, we sought to investigate the functional relevance of this interaction, by exploring the gene expression of candidate genes in stably-transfected HeLa cells expressing the scFv-58F-NLS or the control intrabody scFv-645-NLS. At first, we measured the expression of transcripts that are human orthologs of those most significantly regulated in yeast cells expressing scFv-58F, as indicated by our transcriptome analysis (Figure 1A). However, no significant expression changes in the investigated genes were found (Figure S3A), which might be explained by differences in both the genomic localization and function of H3K9ac between mammalian and yeast cells. Then, we analyzed a number of genes whose expression had been shown to be regulated by HATi treatment, or by knock-down of the GCN5/PCAF complex, in HeLa cells, in previous reports [30,31,34]. Those studies globally modulated histone acetylation with strong alterations in the expression of a subset of genes. Among these, qPCR analysis revealed that SMAD3 (SMAD Family Member 3), SP2 (Sp2 Transcription Factor) and MKNK2 (MAPK Interacting Serine/Threonine Kinase 2) were significantly down-regulated in the presence of scFv-58F-NLS, with respect to WT cells (Figure 4C). Those genes are involved in regulation of transcription (SP2 and SMAD3), in the transmission of signals from the cell surface to the nucleus (SMAD3), or in the mitogen-activated protein kinase signaling pathway being important for translation, cell proliferation, and oncogenic transformation (MKNK2). On the other hand, other transcripts impacted by HATi treatment were, instead, not altered by expressing scFv-58F-NLS (Figure S3B). This finding reinforces the concept that also in human cells the intrabody scFv-58F-NLS allows a more specific transcriptional modulation than that achieved by the HAT inhibitors or by interfering with GCN5/PCAF through precisely targeting H3K9ac. 

Altogether, our results indicate that the intrabody scFv-58F-NLS can be successfully expressed in mammalian cell systems in vitro and that upon expression in the nucleus it induces specific transcriptional regulation of target genes.

## 3. Discussion

Post-translationally modified proteins represent a huge and untapped source of biologically important and disease-relevant targets, but their systematic biological elucidation and validation for research and therapeutic purposes is hampered by a lack of specific tools and experimental strategies. In particular, histone post-translational modifications (PTMs) play a pivotal role in the epigenetic regulation of gene expression [1,2], mediated by proteins known as “writers”, “readers”, and “erasers” [4]. Due to their relevant activity in physiology and pathology, several chemical compounds have been developed to inhibit the function of these proteins, also in a therapeutic perspective [6,7,8]. However, the pleiotropy and multi-specificity of these classes of proteins represent an intrinsic weakness of current epigenetic drugs [14].

Ideally, rather than inhibiting the writer enzymes (e.g., a HAT), that act on several different targets, one would need to target a single PTM site on a given protein per se (that we called “epigenetic word”) [23,35]. We have recently shown the feasibility of an experimental strategy of directly targeting single epigenetic marks with PTM-specific intrabodies [22]. In Chirichella et al. [22], we selected an anti-H3K9ac single-chain variable fragment (scFv-58F) intrabody using the P.I.S.A technology. Importantly, we showed that this intrabody specifically and directly binds the H3K9ac epitope exerting significant functional consequences on transcription and we provided evidence of such interference in living yeast cells. This PTM-specific interference was predicted to achieve a superior specificity and selectivity with respect to the currently used inhibitors targeting the enzymes installing the PTM, but a direct comparison between the two strategies is lacking. Here, we tested this prediction by exploiting a specific anti-H3K9ac single-chain variable fragment (scFv) intrabody, called scFv-58F, recently selected by P.I.S.A technology and shown to induce a functional in-cell interference of the acetylated H3, by selectively binding to acetylated histone H3 in cells [22].

We provide here the first comparative evidence that the direct interference with a single acetylated residue, obtained by a PTM-specific intrabody, results in a remarkably more restricted and specific effect on gene modulation, compared to that achieved by a broader interference using current HAT-targeting small molecule inhibitors. 

The general implication of this result, from a methodological point of view, is that the systematic selection of anti-PTM intrabodies and their use for a PTM-specific interference in living cells will provide a new level of precision and specificity in the description of epigenetics, offering new tools for research and paving the way to new therapeutic opportunities. 

As for the specific PTM analyzed here, namely the acetylated lysine 9 of H3, the greater specificity of the H3K9ac-specific interfering intrabody over standard HATi drugs allowed to demonstrate the transcriptional regulation of genes involved in ribosome biogenesis and function, as a likely downstream target of H3K9 acetylation in *Saccharomyces cerevisiae*. Links between H3K9 acetylation and the modulation of genes involved in ribosome function and biogenesis in yeast have been previously reported, on the basis of systematic mutagenesis of the acetylated lysines [31] or via ChIP and ChIP-seq analyses [32,33,36]. The latter studies established that induction of histone acetylation (and notably of H3K9 acetylation) occurs at several “growth-related genes”, upon stimulation of yeast cells into the growth phase. The genome-wide assessment of H3K9 acetylation locations by ChIP-seq analysis, showed that H3K9 acetylation was present almost exclusively at the promoters of “growth-related genes” (particularly, genes involved in ribosome function and biogenesis, translation, and in amino acid metabolism) to enable their transcription [32,36]. Accordingly, we compared the genes modulated by scFv-58F expression with the genes identified by H3K9ac ChiP-Seq after yeast growth induction [36]. We found that 50 out of 89 genes specifically modulated by scFv-58F (Figure 2B) had been identified as “growth-related” genes in previous work [36], by elevated H3K9ac ChIP-seq signal on their promoters. Moreover, 49 out of the 50 “growth-related” genes modulated by scFv-58F were downregulated, reinforcing the hypothesis that the H3K9ac-binding intrabody acts by inhibiting transcription of a specific subset of genes in yeast (i.e., 84%, 49/58, genes downregulated by scFv-58F, identified as “growth-promoting”).

These comparative data with the literature provide an independent validation of the PTM-selective interference method demonstrated in this paper and show that the superior precision of this method can generate new valuable data and hypotheses for future investigations. As expected, HATs inhibition by the two drugs affected the expression level of a multitude of genes, with Curcumin displaying a larger effect, in line with the literature. The HAT inhibitory activity of Curcumin is principally exerted on p300/CBP, even though it has pleiotropic [29], often contrasting, effects. Indeed, Curcumin has also been reported to inhibit HDAC enzymes, even more potently than other HDAC inhibitors, such as valproic acid and sodium butyrate [37]. Moreover, Curcumin activities both in DNA methyltransferases inhibition [38] and in regulation of miRNA expression [39,40] have been reported. Such broad biological activities underlie the massive transcriptional dysregulation observed after Curcumin treatment.

On the other hand, CPTH2 is a synthetic small-molecule with a more specific inhibitory activity on Gcn5 [24]. This lysine acetyltransferase enzyme is known to acetylate histone H3 lysine 9 [25,41], in addition to other non-histone targets, such as the tumor suppressor p63, the c-Myc oncoprotein, the NF-kB transcription factor [42], and the metabolic co-activator PGC-1alfa [43]. Accordingly, the NGS data analysis shows that CPTH2 yields a more limited effect on gene expression compared to Curcumin. On the other hand, CPTH2 displays a significantly broader effect on gene expression than that observed in the yeast cells expressing the scFv-58F intrabody. It is remarkable that the genes specifically modulated by scFv-58F expression in great part (~70%) overlap with those modulated by CPTH2 treatment, despite representing a minor fraction of the latter (4.2%) (Figure 2C). Interestingly, inspection of the scFv-58F DEGs not overlapping with CPTH2 DEGs (27 genes) showed they encode for proteins involved in processes such as rRNA/ncRNA processing and metabolism. 

To add on to the work in yeast cells, we performed the first steps to evaluate whether the scFv-58F could similarly interfere with transcriptional regulation in mammalian cells. As a first approach, we tested the expression of human orthologs of the genes more strongly downregulated in yeast cells expressing scFv-58F. However, we did not observe significant expression changes for those gene candidates, likely due to differences in either the genomic localization or the function of H3K9ac between mammalian and yeast cells. With the aim of identifying a set of genes potentially susceptible to promoter-localized H3K9ac levels, we therefore analyzed the expression of a set of candidate genes whose expression in HeLa cells had been previously shown to be regulated either by HATi treatment (i.e., Garcinol) [34], or by knock-down of the GCN5/PCAF complex [30]. Among these, a number of candidates were indeed found to be regulated by scFv-58F in HeLa cells: scFv-58F expression in HeLa cells resulted in down regulation of selected genes coding for proteins involved in transcriptional regulation and signaling (i.e., SP2; SMAD3) and in cell proliferation (MKNK2). The results provide the foundation for a more thorough genome-wide analysis of the effects of scFv-58F on gene expression in mammalian cells, which is however out of the scope of the present study and should be the object of further investigation. Nevertheless, these results indicate the ability of scFv-58F to modulate gene expression also in non-yeast cells, opening novel avenues for future studies in mammalian systems.

Altogether, these data have fully supported the prediction that targeting a specific PTM yields more specific downstream effects than inhibiting the enzyme that installs that particular PTM, reflecting the conceptual difference of the two approaches; while the enzyme inhibitors determine a broad cascade of downstream effects, due to the pleiotropic activity of the enzyme on different histone residues and on many non-histone substrates [15,44], the intrabody selectively acts on one particular histone post-translationally modified residue (H3K9ac). Thus, while CPTH2 or Curcumin act by inhibiting broadly acting *writers*, the scFv-58F intrabody targets directly a single and specific *word*.

From a mechanistic point of view, we envision that the first and most likely action of the H3K9ac-specific scFv-58F intrabody is to competitively block the access of H3K9ac readers onto the K9 acetylated H3 protein (either chromatin-engaged or free). In this respect, the H3K9ac scFv-58F antibody domain works like a competing “*reader*” protein, a Chromatin Reader Antibody (CRA) module, devoid of the effector functions of a natural reader. Thus, CRAs are a new class of molecules selective for specific chromatin marks, that can be used as modular building blocks to build combinatorial marks readers (e.g., AND gate, OR gate) or readers with novel effector functions. Besides occluding the acetylated K9 on H3 histone, the scFv-58F antibody might prevent the release of acetyl groups from histones as acetate, thereby enabling subsequent acetylation. Several studies, in fact, have suggested the ability of acetate to influence histone acetylation in mammalian and yeast cells [36].

In recent years, reader inhibitors have been developed (e.g., the BET-bromodomain inhibitor JQ1 [45]). However, BET-bromodomains show considerable target promiscuity [46]. Therefore, any BET-bromodomain inhibitor will intrinsically have pleiotropic effects and henceforth determine broad downstream effects on global gene expression. In any case, a comparison of the H3K9ac scFv-58F intrabody with BET-bromodomain inhibitors, in future work, similarly to what has been reported here with HATi, will be undoubtedly informative. In this respect, one significant advantage of the intracellular antibody P.I.S.A platformis that the specificity and selectivity of the intrabody toward the target PTM-harboring protein depends only on the stringency of the selection and counterselection procedures [22] and, in case of need, could be further improved by directed evolution [47,48].

From a methodological point of view, the general implication of the results presented is that the systematic selection of anti-PTM intrabodies, and their use for a PTM-specific interference in living cells, will provide a new level of precision and specificity in the description of epigenetic processes, offering new tools for research and paving the way to new therapeutic opportunities.

The potency and versatility of the anti-PTM approach relies, first of all, on the universal, diverse, and modular nature of antibody domains. The PTM-binding antibody moiety can be further implemented and tailored by adding suitable effector functions to the H3K9ac binding moiety [23]. This would allow, for instance, targeted degradation of a post-translationally modified pool of a target protein of interest, leading to its specific depletion from the cell [49,50], while sparing the non PT-modified pool. In addition, one could envisage engineering the PTM-specific intrabody to recruit actuators for chromatin retargeting in living cells, similar to an engineered Chromatin Reader (eCR) [51], or for live imaging of the PTM pool of the protein [52].

The H3K9ac-selective scFv-58F intrabody (and other anti-histone PTM-selective intrabodies), delivered via a viral vector, and possibly in a cell-type specific manner, could be used to implement a genetically encoded chromatin immunoprecipitation (intra-ChIP), coupled or not to sequencing (intra-ChIP-seq). This would represent a significant advancement, allowing, for instance, for the performance of cell-type specific ChIP or ChIP-seq from a heterogeneous tissue, such as the brain. Finally, PTM-specific proteomic studies will greatly benefit from the ability to target intracellularly the PTM-pool of a target protein, by fusing for instance the PTM-specific antibody moiety to biotin ligase, for proximity biotinylation, or to TAP (tandem affinity purification) tags, for Affinity Purification mass Spectrometry [53].

In a therapeutic perspective, anti-PTM intrabodies could be delivered as genes or, alternatively, as protein macrodrugs delivered by cell penetrating peptides or by nanocarriers [23]. PTM intrabodies could also provide structural templates to select or to design the first generation of chemicals specifically targeting PTMs [54].

In conclusion, in this study we have validated the PTM Chromatin Reading Antibody approach, as a new strategy to address challenging questions in epigenetic research. Histone modifications are key components of chromatin packaging but whether they constitute a ‘code’ is still debated: are histone modifications causally responsible for differences between chromatin states [55]? Answering this question has proven difficult because of the lack of suitable methods to address causality. Indeed, the functional study of individual histone PTMs presents formidable challenges, and in most organisms can only be indirect. For instance, gene-editing approaches in mammals and other metazoans are extremely demanding because canonical histones are encoded by multiple genes, making it difficult to selectively interfere with specific PTMs [56]. This paper provides a proof of concept for a new method to address these questions. 

Post-translational-specific intrabodies, and in particular acetylation site-specific intrabodies, represent novel specific pharmacological candidates for a variety of research and clinical applications (e.g., in cancer and neurological diseases).

## 4. Materials and Methods

Plasmids and constructs. The coding sequence of the scFv-58F intrabody [22] was cloned in the pLinker220 (pL220) plasmid [57], with the HA tag at its C-terminal. An empty pL220 vector with the HA tag was prepared as a control plasmid as follows. Two complementary primers with the HA sequence with the BssHII and BamHI sites at the 5′ and 3′-terminal, respectively (Forw: 5′ AGCCGAGCGCGCATTACCCTTATGATGTGCCAGATTATGCTTGAGGATCCCCGGG 3′; Rev: 5′ CCCGGGGATCCTCAAGCATAATCTGGCACATCATAAGGGTAATGCGCGCTCGGCT 3′) were in vitro annealed (95 °C for 5 min, followed by a temperature gradient from 95 °C to 25 °C with a 2 °C decrease every minute). The annealed product was BssHII and BamHI digested and cloned in pL220 plasmid.

A second control plasmid was obtained by cloning the coding sequence of an unrelated intrabody (named scFv-645 anti-*Mus musculus* Neuroligin 2, see sequence details in the Supplementary Material), with the HA tag fused at its C-terminal, in pL220.

**Yeast strains.** All experiments have been performed using the *S. cerevisiae* L40 yeast strain characterized by this genotype: mat-a hisΔ200trp1-901 leu2-3,112 ade2 LYS2::(lexAop)4-HIS3 URA3::(lexAop)8-lacZ Gal4 [58]. The pL220 plasmids carrying the scFv-58F-HA, the scFv-645-HA, or the HA tag only, were transformed in the L40 yeast strain, following a previously published LiAc transformation protocol [59].

**Drugs.** Curcumin (Sigma #78246, Merk Life Science S.r.l, Milan, Italy) and CPTH2 (Sigma #C9873, Merk Life Science S.r.l, Milan, Italy) were solubilized in DMSO and used as HAT inhibitors. Cell viability was assessed in pL220–HA yeast strain by growing the cell in SD-L media with increasing concentration (0–800 μM) of the two drugs for 4 or 8 h by counting viable cells (*trypan blue* dye exclusion test). See survival curves in Supplementary Materials (Figure S4).

**Yeast protein extract.** Five experimental conditions have been considered: two drug-treatment conditions (Curcumin or CPTH2), two intrabody-expressing conditions (scFv-58F or scFv-645) and one control condition (yeast strain carrying the empty vector). For each condition, three biological replicates have been considered. All the yeast cells have been treated with the same concentration of 0.4% DMSO. Treatment with Curcumin (200 μM), CPTH2 (400 μM), or control DMSO (0.4%) was performed using the pL220–HA yeast strain, incubated in SD-L medium for 1 h in shaker (250 rpm) at 30 °C. In order to achieve an appropriate metabolic activation of yeasts, two subsequent overnight growths in SD–L medium were performed, before starting the experiment. An aliquot from each sample was taken before and after the hour of incubation, and total protein extract was prepared by Tri-Chloro Acetic Acid (TCA) precipitation. Briefly, 1.5 mL of cell cultures were pelleted and resuspended in 50 µL of lysis buffer (1.85 N NaOH, 7.4% β-mercaptoethanol, 1X Protease Inhibitors Cocktail (Roche #11836170001, Merk Life Science S.r.l, Milan, Italy) for 10 min at RT. A total of 50 µL of cold 50% TCA (Sigma #T6399, Merk Life Science S.r.l, Milan, Italy) were added and incubated in ice for 10 min. Samples were centrifuged at 14,000 rpm for 10 min at 4 °C. The pellets were washed with 110 µL of cold 90% Acetone incubating at −20 °C for 20 min. Samples were centrifuged at 14,000 rpm for 10 min at 4 °C, the pellets resuspended in 40 µL of Sol. A (0.5 M Tris base, 5% SDS) were briefly sonicated, and 40 µL of Sol B (75% glycerol, 1.92% DTT, 0.05% bromophenol blue) was added. Samples were incubated at 95 °C for 10 min, spun, and 10 µL was loaded on acrylamide gel to perform SDS-PAGE.

**Western blot.**

For western blot analysis the following antibodies were used. Total H3 or acetylated H3 were detected using anti-Histone H3 Rabbit Polyclonal Antibody (1:1000; Abcam #1791, Prodotti Gianni, Milan Italy) and anti-acetyl-Histone H3 Rabbit Polyclonal Antibody (1:10,000; EMD Millipore #06-599, Merk Life Science S.r.l, Milan, Italy)), the HA tag using anti-HA (Roche # 11867423001, Merk Life Science S.r.l, Milan, Italy). For loading control of yeast protein samples, anti-PGK Mouse monoclonal (1:10,000; Abcam #22C5D8, Prodotti Gianni, Milan Italy) was used. As secondary antibodies, Anti-Rabbit-HRP (1:2000; Santa Cruz Biotechnologies #sc2004, D.B.A. Italia S.r.l, Segrate (MI) Italy), anti-Mouse-HRP (1:5000; Santa Cruz Biotechnologies #sc-2005, D.B.A. Italia S.r.l, Segrate (MI) Italy), and anti-Rat-HRP (1:2000; Santa CruzBiotechnologies #sc-2006 D.B.A. Italia S.r.l, Segrate (MI) Italy) were used. Chemiluminescence was acquired through a Chemidoc XRS instrument (Bio-Rad Laboratories S.r.l, Segrate (MI) Italy).

**RNA extraction.** An aliquot of the same samples, of the five experimental conditions described above, used for yeast protein extract was used for total RNA isolation. Briefly, after the 1 h of growth, the yeast cultures were centrifuged (5 min at 3000 rpm), and the RNA extraction was performed using the Yeast Ribopure^®^ kit (Ambion, Invitrogen #AM1926, Thermo Fisher Scientific, Rodano (MI) Italy), following manufacturer instructions. The integrity of RNA was assessed by agarose electrophoresis.

The fifteen RNA samples have been delivered to the Leibniz Institute on Aging (FLI) in Jena, where library preparation and sequencing steps have been performed.

**RNA sequencing.** Sequencing of RNA samples was performed using Illumina’s next-generation sequencing methodology [60]. In detail, total RNA was quantified using Agilent 2100 Bioanalyzer Instrument (Agilent RNA 6000 Pico, Agilent Technologies Germany GmbH & Co. KG, Waldbronn, Germany). Libraries were prepared from 1000 ng of input material using TruSeq Stranded mRNA (manufacturer’s instructions) and subsequently quantified and quality checked using Agilent 2100 Bioanalyzer Instrument (DNA 7500 kit). Libraries were pooled and sequenced in one lane of HiSeq 2500 System running in 51 cycle/single-end/high output mode. Sequence information was converted to FASTQ format using bcl2fastq v1.8.4.

**RNA-seq data analysis.** Data were released as FASTQ files and then processed using Linux-based software tools. An initial quality check of the raw sequence data was performed using *FASTQC.* Trimming was performed with the *cutadapt* tool. Using the 3rd version (April 2011) *S. cerevisiae* genome as reference genome, the mapping procedure was carried out with *segemehl* software. The number of mapped reads within the coding regions of each gene was calculated with the *bamutils* software tool (NGSutils suite).

The raw counts statistical analysis (i.e., PCA and hierarchical clustering), the differential expression analysis with the *DESeq2* package, as well as the subsequent statistical analysis (i.e., MA plots and heatmaps), were all performed in R. Gene Ontology enrichment analysis was performed using the ClusterProfiler package in R.

**Real-time qPCR.** After an initial treatment with DNAse (1 h at 37 °C), 400 ng of RNA for each yeast sample have been retro-transcribed using the AMV-RT (Promega #5101, Promega Italia S.r.l, Milano, Italy) and oligo-(dT)_15_ primers. The following TaqMan assays (Thermo Fisher Scientific, Rodano (MI) Italy) have been used: TUB1 (#Sc04175846_s1), URA7 (#Sc04099112_s1), MAK16 (#Sc04097538_s1), SPG1 (#Sc04127525_s1), and SNZ1 (#Sc04154021_s1). For RNA samples from HeLa cells, the following Taqman inventoried assays have been used: ACTB (#Hs99999903_m1), SMAD3 (#Hs00969210_m1), SP2 (#Hs00175262_m1), MKNK2 (#Hs00179671_m1), MAK16 (Hs00261283_m1), POLR1E (#Hs00223953_m1), CTPS2 (#Hs00219845_m1), POLR1D (#Hs04935592_m1), GTPBP4 (#Hs01057434_m1), NAT9 (#Hs07287245_m1), CEBPZ (#Hs00172900_m1), CTPS1 (#Hs01041852_m1), POLR3C (#Hs00197744_m1),TALDO1 (#Hs00997203_m1), CNPPD1 (#Hs01081797_m1), ERRFI1 (#Hs00219060_m1), and BAZ1A (#Hs01056564_m1). Real-time PCR reactions were run in 96-well plates (Bio-Rad Laboratories S.r.l, Segrate (MI) Italy)), using the Step-OnePlus real-time PCR system (Applied Biosystem Italia, Monza, Italy) following this protocol: 2 min at 50 °C, 10 min at 95 °C (Taq-pol activation), followed by 40 cycles with 15 sec at 95 °C (denaturation) and 1 min at 60 °C (annealing and extension). Quantitative values for cDNA amplification were calculated from the threshold cycle number (Ct) obtained during the exponential growth of the PCR products. Threshold was set automatically by the Step one software. Data were analyzed by the ΔΔCt methods using Tubulin (TUB1 gene) for yeast RNA samples or beta Actin (ACTB gene) to normalize the cDNA levels of the transcripts under investigation.

**HeLa stable cell lines for scFv-58F and scFv-645 Immunoprecipitation.**

The scFv-58F was cloned in the pEF1α-IRES-ZsGreen1 Vector (Clontech #631976, Diatech Lab Line S.r.l, Jesi (AN), Italy) inserting a BssHII restriction site at the 5′ prime end and an HA tag at the 3′ prime end (for cytoplasmic expression (58Fcyto) or three nuclear localization signals (NLS) before the HA tag at the 3′ prime end for nuclear expression (58FNLS). The scFv-645 was also cloned in the same vector with the three nuclear localization signals (NLS) before the HA tag at the 3′ prime. 

HeLa cells were plated in 6 well plate (400,000/well), and the next day they were transfected with pEF1α-scFv-58F-HA-IRES-ZsGreen1 or pEF1α-scFv-645-HA-IRES-ZsGreen1, using the Effectene Transfection Reagent (Qiagen # 301427, Qiagen Italia, Milano, Italy). Briefly, 400 ng of the plasmid DNA were diluted in 100 µL of buffer EC and then 3.2 µL of Enhancer were added and incubated at RT for 5 min. Then, 10 µL of Effectene were added and the solution mixed by vortex for 10 s and incubated at RT for 20 min to allow transfection complex formation. Meanwhile, cells were washed once with PBS and 1.6 mL fresh growth medium (DMEM + 10%FBS penicillin/streptomycin 100 U/mL) were added to the cells. At the end of the 20 minutes, 600 µL of fresh growth medium were added to the transfection complexes and immediately added dropwise onto the cells in the 6-well plates. The next day the media was changed with fresh growth medium containing 1 mg/mL geneticin (G418) (Euroclone # ECM0015C, Euroclone S.p.A, Pero (MI)). Cells were FACS sorted for the green fluorescent protein ZsGreen1 48 h post transfection. Pooled green fluorescent cells were kept in culture with the selective geneticin medium for the following 4 weeks, and pooled stable transfected cells were generated.

**Immunoprecipitation.**

For nucleosome extract preparations, HeLa cells cultured in Petri dishes (100 × 15 mm) were trypsinized and washed in PBS. The pellet was resuspended in RSB buffer (10 mM Tris-Cl pH 7.4, 10 mM NaCl, 3 mM MgCl_2_). The cells were left on ice and after 10 min RSB buffer + 1% NP40 was added to the samples. Samples were centrifuged, and the resulting nuclei were washed twice with PBS. Nuclei were resuspended in PBS and 1% formaldehyde was added to crosslink the chromatin. The reaction was blocked by adding 125 mM glycine. Nuclei were centrifuged at a low speed, and washed with PBS and 1 M NaCl. After further washing with PBS, the nuclei were resuspended in MNase buffer (100 mM Tris-Cl pH 7.5, 50 mM NaCl, 8 mM MgCl_2_, 2 mM CaCl_2_) and left on ice for 30 min. MNase (Roche #10107921001) was added to the solution (final concentration 400 U/mL) and incubated at 37 °C for 1 h and 30 min. The reaction was stopped by adding 7.5 mM EDTA. The samples were diluted in a 2X dilution buffer (250 mM NaCl, 2 mM EDTA, 0.2% NP40, 0.4% SDS). The samples were left on ice for 10 min, sonicated for 10 cycles 2 s ON + 10 s OFF, medium power, and centrifuge at 14,000× *g*, 10 min at 4 °C. The supernatant was collected and quantified.

A total of 500 ug of extracts were used for each immunoprecipitation (IP). Before IP, the samples were precleared with 20 µL of Protein G beads (Santa Cruz) and BSA. The beads for IP were pre-incubated with BSA, and the sample was used for buffer resuspension. The IP was performed adding to each pre-cleared sample 2 ug of specific antibody: anti-acetyl-Histone H3 (Millipore # 06-599, Merk Life Science S.r.l, Milan, Italy) and anti-Histone H3 (Abcam #1791, Prodotti Gianni, Milan Italy), in rotation at 4 °C for 5 hs. To monitor the specificity of ChIP assays, samples were also immunoprecipitated with a specific-antibody isotype matched control immunoglobulin (IgG). Then, 35 µL of Protein G beads were added and samples were put to rotate O/N at 4 °C. In the morning, beads were recovered, sequentially washed in a low salt buffer, high salt buffer, LiCl buffer, and RIPA. Finally, the beads were resuspended in 30 µL of 2X laemmli buffer, boiled for 10 min, and the eluates were analyzed through 15% SDS-PAGE, as detailed in the western blot paragraph.

**RNA extraction for qPCR from Hela Cells.**

HeLa cells were washed once in PBS, and collected by adding Phenol/guanidine-based QIAzol Lysis Reagent (Qiagen #79306, Qiagen Italia, Milano, Italy) to the plate. The samples were put in 2 mL tubes, chloroform was added, and the samples were shaken for 15 s. The samples were left at 20–24 °C for 3 min and then centrifuged (12,000× *g*, 20 min, 4 °C). The upper phase aqueous solution containing RNA was collected in a fresh tube, and the RNA was precipitated by the addition of isopropanol. Samples were mixed by vortexing, left at RT for 15 min and then centrifuged (12,000× *g*, 20 min, 4 °C). The supernatant was discarded and the RNA pellet was washed in 75% ethanol by centrifugation (7500× *g*, 10 min, 4 °C). The supernatant was discarded and the pellet was left to dry for at least 15 min; then, it was resuspended in RNAse free water. RNA concentration was determined by Nanodrop Spectrophotometer (Thermoscientific 2000 C, Thermo Fisher Scientific, Rodano (MI) Italy). RNA quality was analyzed through a gel running (1% agarose). Total RNA was reverse transcribed using QuantiTech Reverse Transcription Kit (Qiagen # 205311, Qiagen Italia, Milano, Italy). Gene expression was analyzed by real-time PCR (Step-OnePlus real-time PCR system, Applied Biosystem Italia, Monza, Italy), using Taqman inventoried assays as explained in the Real-time qPCR paragraph.

## Data Availability

The data presented in this study are openly available in NCBI’s Gene Expression Omnibus and are accessible through GEO Series accession number GSE194577, accessed on 30 January 2022 (https://www.ncbi.nlm.nih.gov/geo/query/acc.cgi?acc=GSE195547).

## Supplementary Materials

The following supporting information can be downloaded at: https://www.mdpi.com/article/10.3390/ijms23168892/s1.
