# Supplementary material for: Acetylation-Specific Interference by Anti-Histone H3K9ac Intrabody Results in Precise Modulation of Gene Expression
Source: Int J Mol Sci. 2022 Aug 10;23(16):8892. doi: 10.3390/ijms23168892 (PMC9408029; doi:10.3390/ijms23168892)
Supplement: Supplementary file 1 [file ijms-23-08892-s001.zip › Supplemental Information.pdf]

## Supplementary Information for

# Acetylation-Specific Interference by Anti-Histone H3K9ac Intrabody Results in Precise Modulation of Gene Expression

**Simonetta Lisi [1,†], Matteo Trovato [1,2,†], Ottavia Vitaloni [1], Marco Fantini [1], Michele Chirichella [1], Paola Tognini [1,3], Sara Cornuti [1], Mario Costa [4], Marco Groth [5] and Antonino Cattaneo [1,*]**

[1] Bio@SNS Laboratory, Scuola Normale Superiore, 56126 Pisa, Italy
[2] European Molecular Biology Laboratory, Genome Biology Unit, 69117 Heidelberg, Germany
[3] Department of Translational Research and New Technologies in Medicine and Surgery, University of Pisa, 56126 Pisa, Italy
[4] Institute of Neurosciences, Consiglio Nazionale Delle Ricerche, 56124 Pisa, Italy
[5] Leibniz Institute on Aging—Fritz Lipmann Institute (FLI), 07745 Jena, Germany
* Correspondence: antonino.cattaneo@sns.it; Tel.: +39-050-509320
† These authors contributed equally to this work.

**This PDF file includes:**

Supplementary text
Figures S1 to S4
Legends for Datasets S1 to S2
SI References

**Other supplementary materials for this manuscript include the following:**

Datasets S1 to S2

**Supplementary Information Text**

**Aminoacid sequence of scFv645 anti *Mus musculus* Neuroligin 2**
DIVLTQSQKFMSTSVGDRVSVTCKASQNVGTNVAWYQQKPGQSPKALIYSASYRYSGVPDRFT
GSGSGTDYTLTISNVQSEDLAEYFCQQYNSYPLTFGAGTKLEIKRSGGSTSGSGKPGSGEGSSGT
QVQLQQSGAELVRPGASVKMSCKASGYTFTSYWMHWVKQRPGQGLEWIGTIDPSDSYTNYN
QKFKDKATLTVDTSSSTAYMQFNSLTPEDSAVYYCTRSYGSNYAMDYWCQGTTITVSS

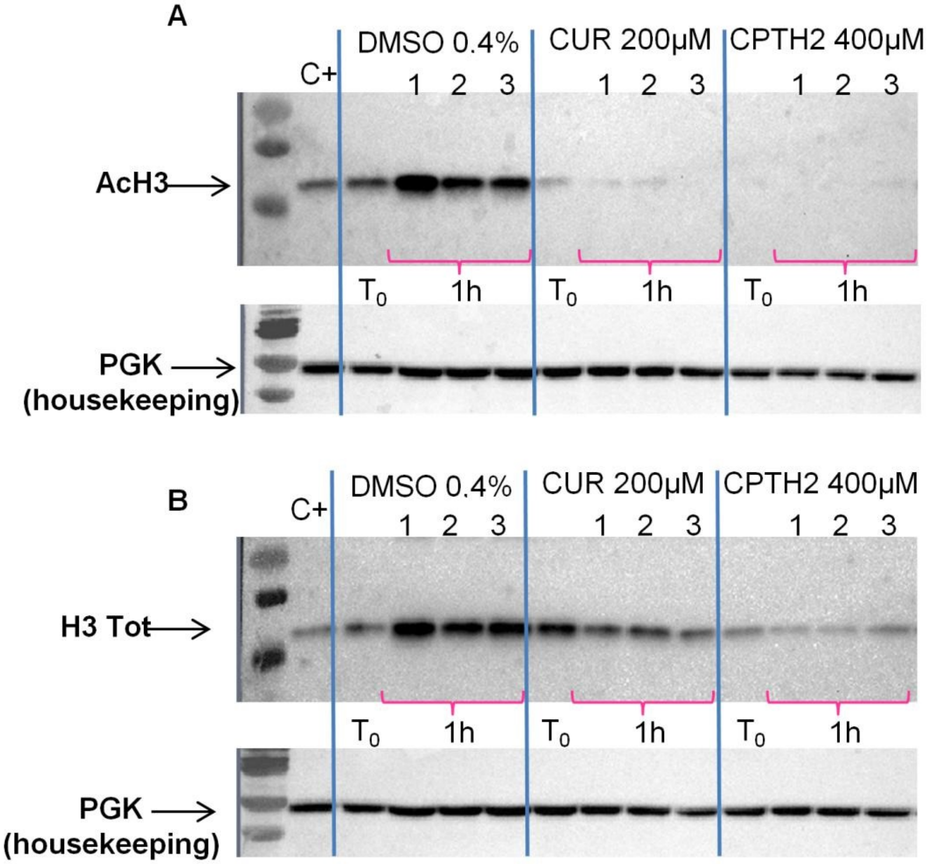


**Figure S1. Western blot of H3 acetylation after HATi inhibition.** A) Acetylated H3 (top panel) and the housekeeping Phosphoglycerate kinase (PGK) (bottom panel) and B) total H3 (top panel) and the housekeeping PGK (bottom panel) in protein extract from the yeast strain pL220-HA treated with Curcumin (200 µM), CPTH2 (400 µM) or DMSO (0.4%) for 1 hour at 30°C. Three biological replicates (1-2-3) are shown for each condition. PGK was used as loading control

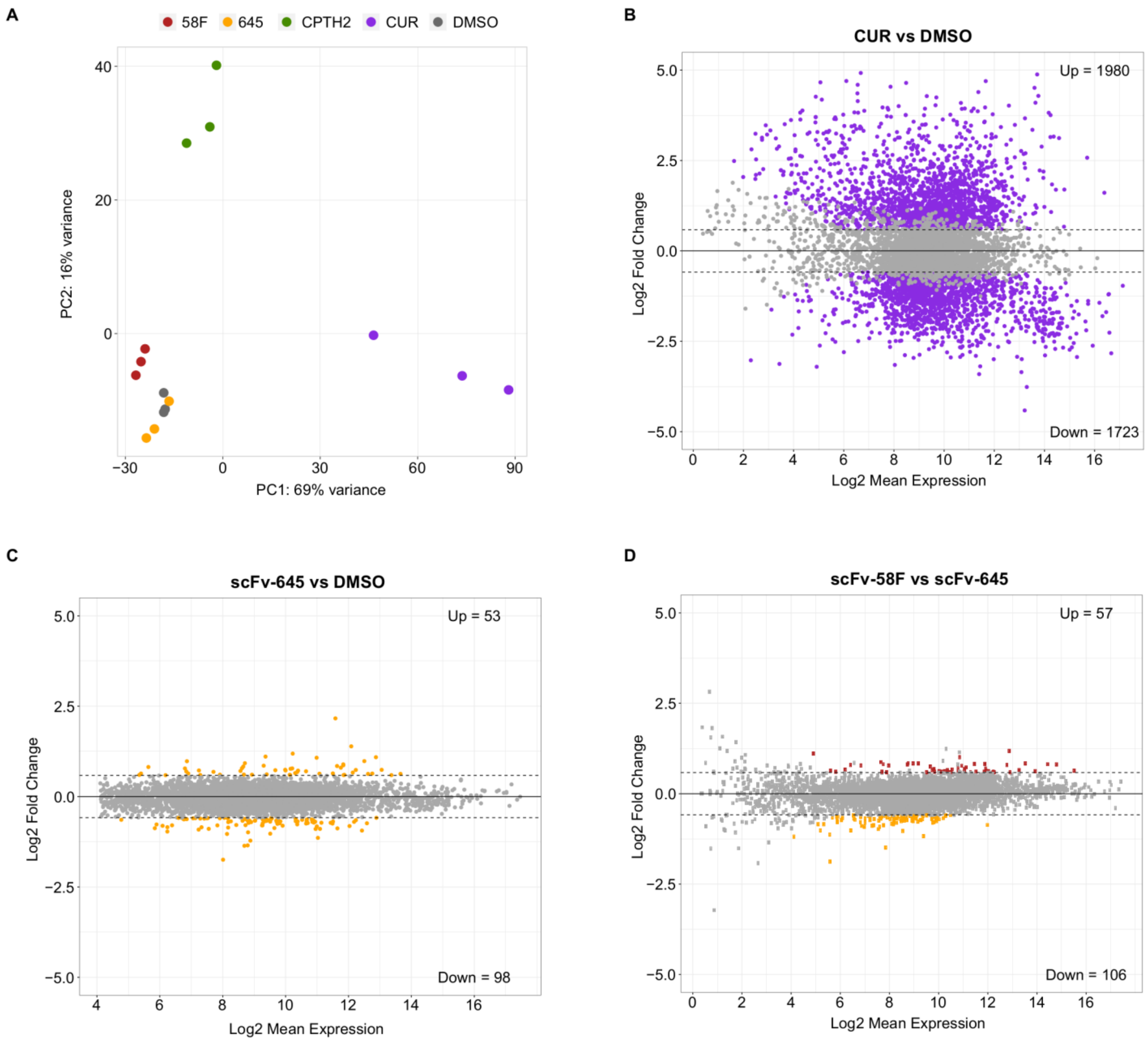


**Figure S2. Expression of scFV-58F intrabody induces fewer transcriptional changes than treatment with HAT inhibitors**. (**A**) PCA plot including all samples. CPTH2 and Curcumin treatments account for most of the variation. (**B-D**) MA plot representing the differentially expressed genes (FDR=0.05) for Curcumin-treated cells (**B**) and yeast cells expressing the scFv-645 intrabody (**C**), versus DMSO-treated cells. MA plot from the differential gene expression analysis between scFv-58F- and scFv-645-expressing yeast cells is also reported (**D**). The number of significantly up-/down-regulated genes (padj<0.05 & FC cutoff=1.5) is reported for all comparisons.

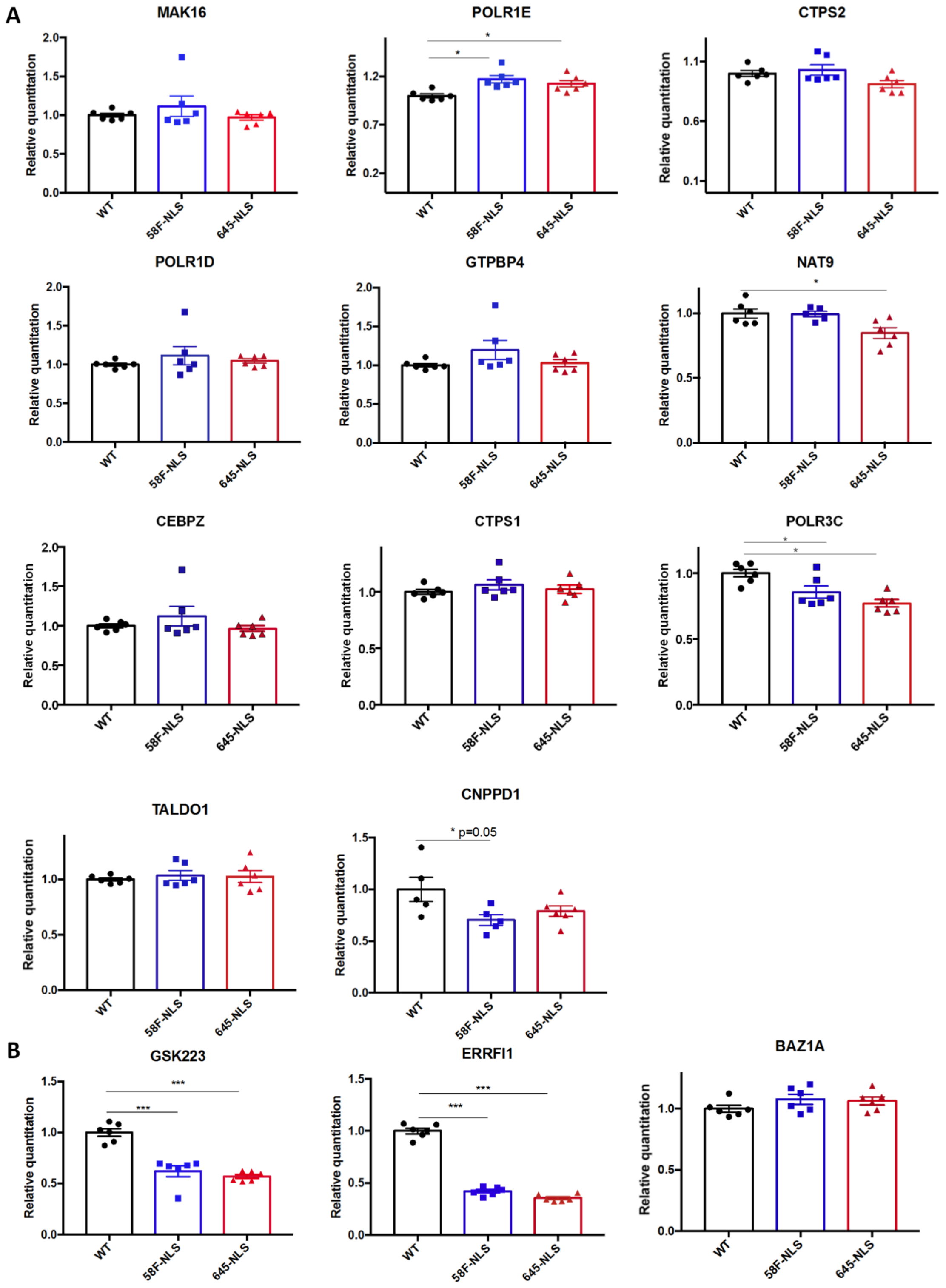


**Figure S3. Gene expression in stable transfected HeLa cells**. RNA was prepared from WT HeLa cells (WT), stable transfected HeLa cells expressing scFv-58F-NLS (58F-NLS), and stable

transfected HeLa cells expressing scFv-645-NLS (645-NLS). **A)** qPCR analysis of genes regulated in the yeast by expression of scFv-58F-NLS was performed. Data is represented as relative RNA levels the transcript of interest normalized to beta actin (ACTB gene). (n=6 per groups, one-way ANOVA, post-hoc Tukey's multiple comparison test: WT vs 58F-NLS, WT vs 645-NLS $p<0.05$*. All other comparisons not significant). **B**) qPCR analysis of genes regulated by HATi treatment in HeLa cells [1,2]. Data is represented as relative RNA levels of the transcript of interest normalized to beta actin (ACTB gene). (n=6 per groups, one-way ANOVA, post-hoc Tukey's multiple comparison test: WT vs 58F-NLS, WT vs 645-NLS $p<0.0001$***. All other comparisons not significant). Error bars represent SEM, dots over the histogram represent individual biological replicate.

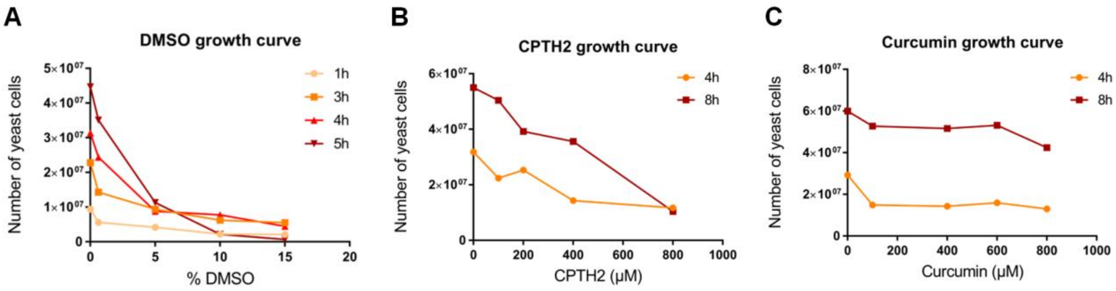


**Figure S4. Yeast strain pL220-HA growth curves in the presence of different concentrations of DMSO, CPTH2 or Curcumin.** For DMSO growth curve (**A**), the effect on yeast viability was assessed by measuring the absorbance at 600 nm at different time points. The number of yeast cells was estimated considering the following conversion factor: $OD_{(600\ nm)}$=1.0 corresponding to $1.4x10^7$ cells. For CPTH2 (**B**) and Curcumin (**C**) growth curves, viability was assessed via trypan blue staining after 4 and 8h of growth.

**Dataset S1 (separate file).** Complete lists of genes (DESeq2 output) referred to analyses comparing either the two HATi-treated or the two intrabody-expressing conditions against the DMSO-treated baseline control condition.

**Dataset S2 (separate file).** List of genes significantly differentially expressed by CPTH2 (FC cut-off=1.5) or scFv-58F, related to Fig.2.

**SI References**


1. Balasubramanyam, K.; Altaf, M.; Varier, R.A.; Swaminathan, V.; Ravindran, A.; Sadhale, P.P.; Kundu, T.K. Polyisoprenylated Benzophenone, Garcinol, a Natural Histone Acetyltransferase Inhibitor, Represses Chromatin Transcription and Alters Global Gene Expression *. *J. Biol. Chem.* **2004**, *279*, 33716–33726, doi:10.1074/JBC.M402839200.
2. Huang, H.; Maertens, A.M.; Hyland, E.M.; Dai, J.; Norris, A.; Boeke, J.D.; Bader, J.S. HistoneHits: A Database for Histone Mutations and Their Phenotypes. *Genome Res.* **2009**, *19*, 674–681, doi:10.1101/GR.083402.108.
